# Characterization and Activity Analyses of the *FLOWERING LOCUS T* Promoter in *Gossypium Hirsutum*

**DOI:** 10.3390/ijms20194769

**Published:** 2019-09-26

**Authors:** Na Sang, Darun Cai, Chao Li, Yuqiang Sun, Xianzhong Huang

**Affiliations:** 1 The Key Laboratory of Oasis Eco-Agriculture, College of Agriculture, Shihezi University, Shihezi 832000, China; sn899097@163.com; 2 Special Plant Genomics Laboratory, College of Life Sciences, Shihezi University, Shihezi 832000, China; 3 State Key Laboratory of Plant Cell and Chromosome Engineering, Institute of Genetics and Developmental Biology, Chinese Academy of Sciences, Beijing 100101, China; cdrhxz104@163.com; 4 State Key Laboratory of Plant Cell and Chromosome Engineering, Center for Genome Editing, Institute of Genetics and Developmental Biology, Chinese Academy of Sciences, Beijing 100101, China; chaoyue_014@163.com; 5 Key Laboratory of Plant Secondary Metabolism and Regulation of Zhejiang Province, College of Life Sciences, Zhejiang Sci-Tech University, Hangzhou 310016, Zhejiang, China; 6 College of Agriculture, Anhui Science and Technology University, Fengyang 233100, Anhui, China

**Keywords:** cotton, *FLOWERING LOCUS T*, promoter analysis, flowering transition, CORE

## Abstract

Flowering transition is a crucial development process in cotton (*Gossypium hirsutum* L.), and the flowering time is closely correlated with the timing of *FLOWERING LOCUS T* (*FT*) expression. However, the mechanism underlying the coordination of various *cis*-regulatory elements in the *FT* promoter of cotton has not been determined. In this study, a 5.9-kb promoter of *FT* was identified from cotton. A bioinformatics analysis showed that multiple insertion–deletion sites existed in the 5.9-kb promoter. Different expression levels of a reporter gene, and the induction by sequential deletions in *GhFT* promoter, demonstrated that 1.8-kb of the *GhFT* promoter was stronger than 4.2-, 4.8-, and 5.9-kb promoter fragments. The binding sites of the CONSTANS (CO) and NUCLEAR FACTOR Y transcription factors were located within the 1.0-kb sequence upstream of the *FT* transcription start site. A large number of repeat segments were identified in proximal promoter regions (−1.1 to −1.4 kb). A complementation analysis of deletion constructs between 1.0 and 1.8 kb of *G. hirsutum*, *Gossypium arboretum*, and *Gossypium raimondii* *FT* promoters revealed that the 1.0-kb fragment significantly rescued the late-flowering phenotype of the *Arabidopsis* *FT* loss-of-function mutant *ft-10*, whereas the 1.8-kb promoter only slightly rescued the late-flowering phenotype. Furthermore, the conserved CORE motif in the cotton *FT* promoter is an atypical TGTG(N2-3)ATG, but the number of arbitrary bases between TGTG and ATG is uncertain. Thus, the proximal *FT* promoter region might play an important role affecting the activity levels of *FT* promoters in cotton flowering.

## 1. Introduction

Flowering in plants is an important physiological process in the switch from vegetative to reproductive growth, and this process is strictly controlled by a complex regulatory network consisting of environmental cues and plant developmental signals [1]. The regulation of the *FLOWERING LOCUS T* (*FT*) gene in *Arabidopsis thaliana* is understood to involve at least six major pathways controlling flowering: the photoperiod, vernalization, ambient temperature, age, autonomous, and gibberellin pathways. They converge at the integrator *FT* gene [2,3]. FT protein is produced in the vascular tissues of leaves and is translocated to the shoot apical meristem, where it can interact with the basic leucine zipper transcription factor FD to form a complex and then activate the expression of downstream genes involved in floral morphogenesis, such as *APETALA1* and *LEAFY* [4,5,6,7,8].

The transcriptional activation of *FT* is directly mediated by CONSTANS (CO) under long-day (LD) inductive conditions [1]. CORE1 and CORE2 are the two CO responsive elements in the *FT* promoter that share the consensus sequence TGTG(N2-3)ATG [9]. The CONSTANS, CONSTANS-like, and TOC1 domain, which is a conserved motif at the C-terminus of CO, can interact with nuclear factor Y (NF-Y) transcription factors [9,10,11]. Cao et al. (2014) proposed a photoperiodic flowering recruitment model: NF-Y complexes bind to CCAAT distal to the *FT* promoter and recruit and stabilize the binding of CO to CORE1 and CORE2 proximal to the *FT* promoter to facilitate the transcriptional activation of *FT* [12]. Upland cotton (*Gossypium hirsutum* L.) is an allotetraploid species, which was formed by hybridization of the A-genome ancestor *Gossypium arboretum* (AA) with D-genome ancestor *Gossypium raimondii* (DD), followed by chromosomal doubling [13]. Homeologous gene pairs are included in the A and D subgenomes. Our group previously identified 42 CO-like (*COL*) homologues in the *G. hirsutum* genome, and a phylogenetic analysis classified them into three groups [14]. Group I contains 14 *GhCOLs*, which are clustered with *AtCO* and rice (*Oryza sativa*) *Hd1* [15]. One homoeologous pair, *GhCOL1-A* and *GhCOL1-D*, in Group I have the greatest sequence similarities with *AtCO* and *Hd1*. Furthermore, the overexpression of *GhCOL1* can fully rescue the late-flowering phenotype of the *co-2* mutant, suggesting that the *GhCOL1* homologues act as flowering inducers in *G. hirsutum* [14].

To clarify the complex relationships between activation and suppression signals that regulate flowering through *FT*, Adrian et al. (2010) studied the conserved sequence of the *FT* promoter in *Arabidopsis* in detail. First, the 7.0-kb *FT* promoter sequences of Columbia (Col), *Landsberg erecta* (Ler), *Arabidopsis lyrata*, *Brassica rapa*, and *Arabis alpine* were compared. Three conserved sequence regions were located upstream, in the middle and downstream of the 7.0-kb *FT* promoter and are known as Blocks A, B, and C, respectively. Further investigations of the *FT* promoter (1.0-, 4.0-, 5.7-, and 8.1-kb segments) showed that Block A, located at the proximal end, and Block C, located at the distal end at approximately 5.0-kb, played important roles in the activity of the *FT* promoter mediated by *CO*. Furthermore, the overexpression of *CO* resulted in changes in the chromatin state, such as decreased LIKE HETEROCHROMATIN PROTEIN1 (LHP1) binding and increased H3K9K14 acetylation. These changes could be the results of the upregulation of *FT* expression, rather than a prerequisite for *FT* activation [16]. Furthermore, a number of other studies on the interactions between *cis*-acting elements on the *FT* promoter and corresponding transcription factors have also been conducted in *Arabidopsis* [17,18,19,20].

The function of the *FT* promoter has been studied in other plants, such as *FT2a* in soybean (*Glycine max*) [21], *Hd3a* in rice [22], and *FT* in wheat (*Triticum aestivum*) [23]. Our group identified a cotton *FT* homologous gene, *GhFT*, from *G. hirsutum* and demonstrated that it was predominantly expressed in stamens and sepals and had a relatively higher expression level during the initiation stage of fiber development. The ectopic overexpression of *GhFT* promoted flowering, lateral shoot growth, and leaf morphology, indicating that cotton *FT* regulates shoot architecture by advancing determinate growth [24,25]. Similar results have been reported by other groups [26,27,28]. However, details of the *FT* promoter in cotton, such as its activity, function, and sequence, remain unknown and require further study. Here, we analyzed the activity of the 5.9-kb promoter of *GhFT* using a series of deletion constructs and found that the *GhFT* promoter had an activity length of approximately 1.8-kb upstream of the transcription start site. Further research is warranted to examine the 1.8-kb promoter in *G. hirsutum*, *G. arboretum*, and *G. raimondii*; therefore, we have constructed a truncated 1.0-kb promoter, and the GUS activity induced by which was stronger and was more efficient in rescuing the late-flowering phenotype of *ft-10* than the 1.8-kb promoter.

## 2. Results

### 2.1. Bioinformatics Analysis of the FT Promoters in Cotton

**Figure 1:**
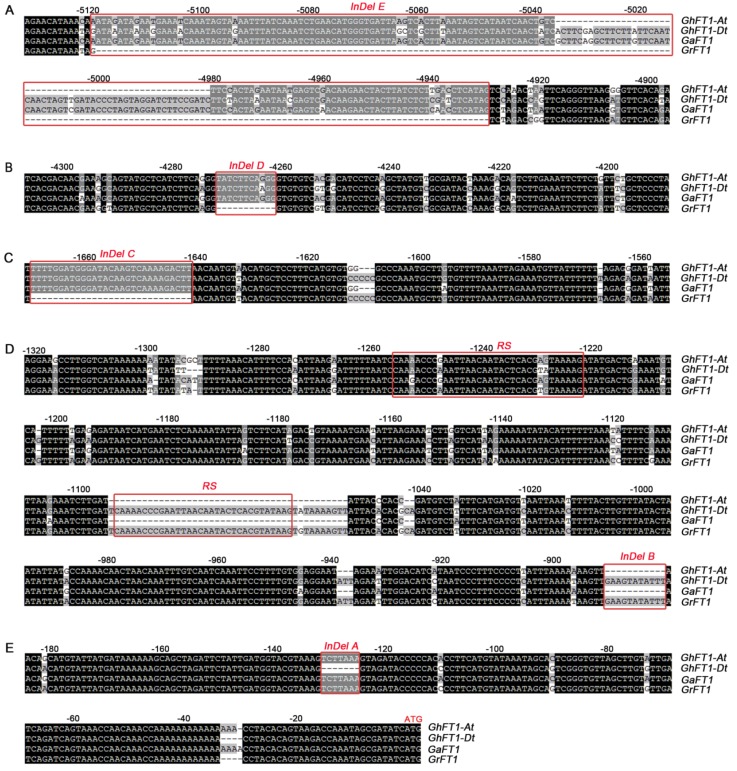
Multiple sequence alignment of the *FT* promoter among *G. hirsutum*, *G. raimondii* and *G. arboreum*. (**A**) Sequence alignment of the distal *FT* promoter (−4900 to −5120 bp). One *Indel* sequence was identified and called *Indel E* (−4930 to −5120 bp). (**B**) Sequence alignment of the *FT* promoter (−4190 to −4305 bp). One *Indel* sequence was identified and named *Indel D* (−4260 to −4270 bp). (**C**) Sequence alignment of the *FT* promoter (−1560 to −1670 bp). One *Indel* sequence was identified and named *Indel C* (−1640 to −1670 bp). (**D**) Sequence alignment of the *FT* promoter (−4190 to −4305 bp). One *Indel* sequence was identified and named *Indel* B (−879 to −890 bp). One repetitive sequence was also found (−1220 to −1260 bp and −1055 to −1095 bp). (**E**) Sequence alignment of a proximal *FT* promoter region (−1 to −185 bp). One *Indel* sequence was identified and named *Indel A* (−879 to −890 bp).

To identify candidate sequences for regulatory motifs, the sequences from 5.9-kb upstream of *FT* in *G. hirsutum*, *G. arboretum*, and *G. raimondii* were analyzed. The pairwise alignment revealed five Indel sites among them (Figure 1). *Indels E*, *D*, and *C* are located approximately 4.9-, 4.2-, and 1.6-kb upstream of the *FT* start codon, respectively, and do not exist in *G. raimondii* (Figure 1A–C)*. Indel B* is located approximately 0.9-kb upstream of the *FT* initiation codon and does not exist in *G. arboreum* or the A subgenome promoters of *G. hirsutum*. Furthermore, repeat sequences (RSs) are identified approximately 1.0-kb and 1.2-kb upstream of ATG, but *G. arboreum* and the A subgenomes of *G. hirsutum* lack these RSs (Figure 1D). *Indel A*, closest to the *FT* start codon, have been deleted in the D subgenome promoter of *G. hirsutum* (Figure 1E).

### 2.2. Histochemical Activity Analysis of GhFT Promoter

**Figure 2:**
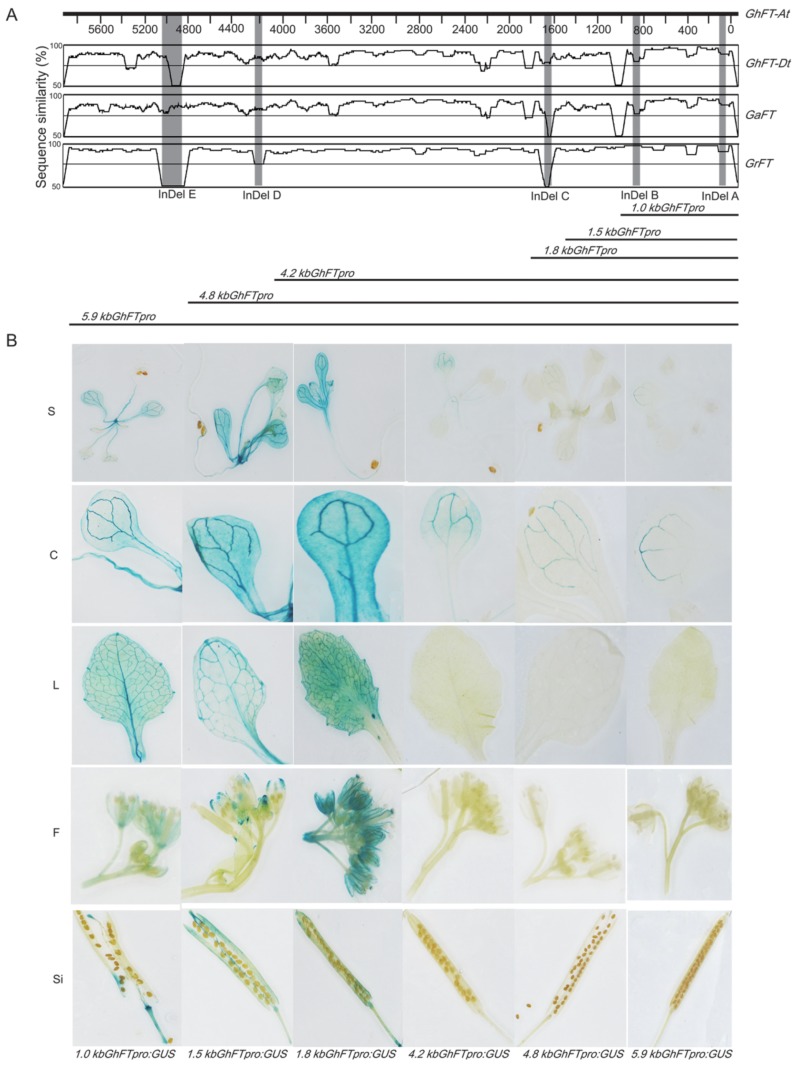
Histochemical activity analyses of the *GhFT* promoter. (**A**) Alignment of the same promoter region of the 5.9-kb *FT* promoter from *G. hirsutum*, *G. arboretum*, and *G. raimondii* using mVISTA. Graphical shows base-pair identity in a range of 50–100%. Light-gray areas showed different Indels. Promoter constructs used for analyses are depicted as black boxes. A transient expression assay was performed using a *GUS* gene under the control of *FT* promoter fragments of 1.0-, 1.5-, 1.8-, 4.2-, 4.8-, and 5.9-kb in length. (**B**) GUS activity analysis of the *GhFT* promoter in transgenic plants of different deletions and different tissues. S, C, L, F, and Si represent seedlings, cotyledons, leaves, flowers, and siliques, respectively.

To assess the activity of the *GhFT* promoter, *GhFT* promoter deletion constructs (1.0-, 1.5-, 1.8-, 4.2-, 4.8-, and 5.9-kb *FT* promoter fragments) were independently fused with GUS (Figure 2A). These constructs were stably transformed into *A. thaliana* Col-0 plants using *Agrobacterium*-mediated genetic transformation. Histochemical GUS staining was performed in various tissues, such as seedlings, cotyledons, leaves, flowers, and siliques (Figure 2B). In seedlings and cotyledons, the GUS activities were induced by all the promoter fragments, and the 1.0-, 1.5-, and 1.8-kb fragments’ activities were higher than those of the 4.2-, 4.8-, and 5.9-kb fragments. The GUS activities induced by the 1.0-, 1.5-, and 1.8-kb fragments were higher in leaves, flowers, and siliques, in which did not induced by the 4.2-, 4.8-, and 5.9-kb fragments. We speculate that the 4.2-, 4.8-, and 5.9-kb promoter fragments may have the ability to induce transcription, but there are some upstream repressing sequences between 1.8- and 5.9-kb, which needs to be confirmed in the future. These results indicated that the activity regulator of the *GhFT* promoter in *G. hirsutum* was approximately 1.8-kb upstream of the *FT* start codon, but it did not exclude the effects of other distal sequences on *GhFT* expression.

### 2.3. Sequence Analyses of the 1.8-kb FT Promoters in G. hirsutum, G. arboretum, and G. raimondii

**Figure 3:**
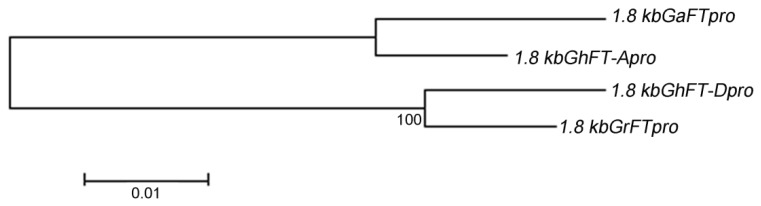
Phylogenetic analyses of the 1.8-kb *FT* promoter of *G. hirsutum*, *G. arboretum*, and *G. raimondii*. The phylogenetic tree was constructed using MEGA5.1. The numbers on the branches represent the reliability percent of bootstrap values based on 1000 replications, and the scale represents nucleotide substitution ratio.

To analyze the 1.8-kb promoter of *FT*, we first performed a phylogenetic analysis of *G. hirsutum*, *G. arboretum*, and *G. raimondii*. The constructed phylogenetic tree indicated that the 1.8-kb cotton *FT* promoters could be clearly divided into two types of the A and D subgenomes on the basis of the source of the promoter genome (Figure 3).

We further amplified the approximately 1.8-kb *GhFT* promoter sequences from the A and D subgenomes of *G. hirsutum*. Sequencing confirmed that the length of the *GhFT-A* promoter was 1771 bp, and the length of the *GhFT-D* promoter was 1701 bp. A bioinformatics analysis by the PlantCare program revealed that these 1.8-kb fragments contain a number of putative plant *cis*-elements. The predicted core promoter elements, such as the TATA-box and CAAT-box, were identified (Tables S1 and S2). In addition, some hormone-related elements were also recognized, including the TCA-element, ABRE, the TATC-box and the P-box. Light-responsive elements, such as the as-2-box, the G-box, the ATCT-motif, ACE, Box1, Box4, Pc-CMA2c, the AT1-motif, the GT1-motif, Sp1 and the TCT-motif, were also observed. There were also abiotic stress-tolerance factors, such as HSE, TC-rich repeats, MBS, and the GC-motif. Among tissue-expression motifs, the CAT-box motif and circadian rhythm-related elements also existed in these two sequences (Tables S1 and S2).

### 2.4. The 1.0-kb Sequence Upstream of the FT Translation Initiation Site Concentrated CORE and CCAAT Domains

Two CO-responsive elements (CORE1 and CORE2) in the *AtFT* promoter share a consensus sequence, TGTG(N2-3)ATG [9]. CO proteins can interact with NF-Y transcription factors [10,11]. NF-Y proteins consist of three unique subunits, NF-YA, NF-YB, and NF-YC, which form a heterotrimeric complex that binds DNA and can recognize *CCAAT cis*-elements [29,30,31]. Because our experiment showed that the strong GUS activities were induced by the 1.8-kb cotton *GhFT* promoter fragment (Figure 2), we next analyzed the CORE and CCAAT domains in this 1.8-kb sequence to predict the CO and NF-Y transcription factor binding sites. The CORE and CCAAT domains were concentrated in the 1.0-kb region upstream of the translation start site, but there was no CCAAT domain between 1.0 and 1.8 kb in the promoter (Figure S1A,B), which was different from in *Arabidopsis* [12]. However, compared with the consensus sequence TGTG(N2-3)ATG reported previously, the conserved motif of CORE in the cotton *FT* promoter is the atypical TGTG(N2-3)ATG, and the number of arbitrary bases between TGTG and ATG is uncertain, being one or six (Figure S1A,B). The FT/CO regulon model is highly conserved in photosensitive plants [32,33,34]. Thus, whether the FT/CO regulatory model is conserved in upland cotton remains to be determined.

**Figure 4:**
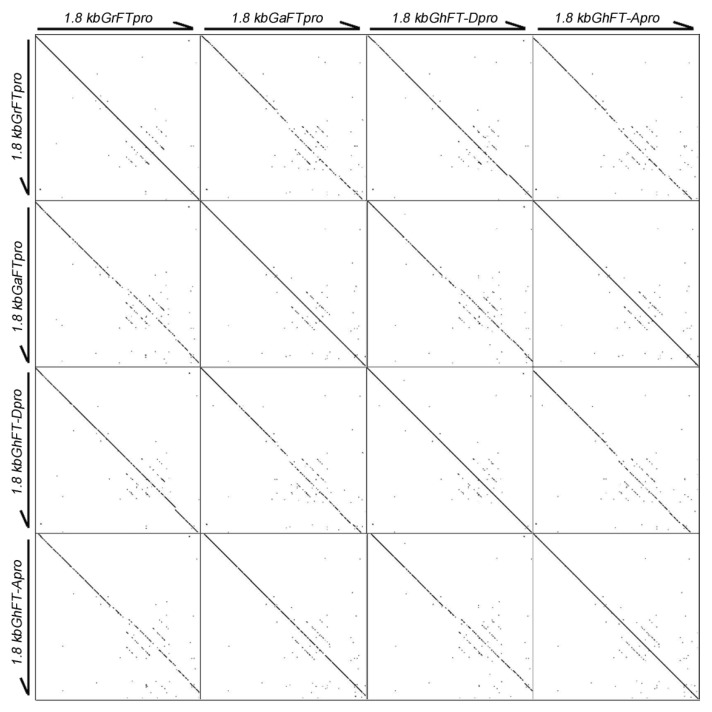
Repeat sequences in the 1.8-kb cotton *FT* promoter in *G. hirsutum*, *G. arboretum*, and *G. raimondii*. Harr-plot was used and the black arrow points downstream of the promoter.

The RSs within the 1.8-kb region upstream of the *FT* start codon in *G. arboreum*, *G. raimondii*, and *G. hirsutum* were further analyzed. We found that a large number of repeated fragments exist between 1.1- and 1.4-kb upstream of the *FT* promoters, where the *cis*-acting elements are also enriched (Figure 4). Thus, these repetitive sequences between 1.1- and 1.4-kb upstream of the translation start site may play important roles in the activity of the cotton *FT* promoter.

### 2.5. Histochemical Activity of the 1.8-kb Sequence Upstream of the FT Promoter in Cotton

**Figure 5:**
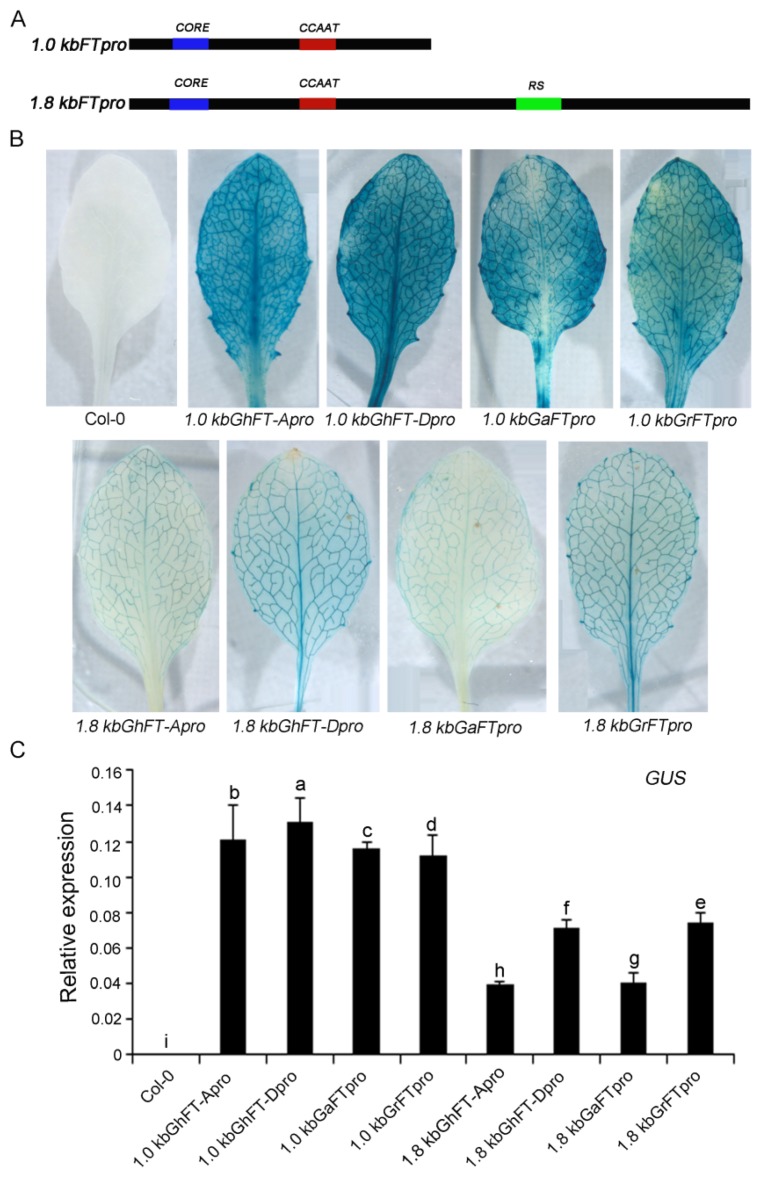
Histochemical activity analyses of the 1.8-kb sequence upstream of cotton *FT* promoters. (**A**) Truncation of the cotton *FT* promoters of *G. hirsutum*, *G. arboretum*, and *G. raimondii*. *RS* represents the domain of enriched repeat sequences. (**B**) The histochemical analysis of GUS activity in the leaves of *Arabidopsis* growing on flowering day; (**C**) The expression level of the *GUS* gene in the transgenic *Arabidopsis* and wild type plants (Col-0) by qRT-PCR. Total RNA was extracted from the true leave tissues of *Arabidopsis* on flowering day. Data represent the mean ± SE (*n* = 3) obtained from three biological replicates. Statistically significant differences are indicated by different lower-case letters (*p* < 0.05, Duncan’s multiple range tests).

We hypothesized that the proximal cotton *FT* promoter (−1 to −1000 bp) may play important roles in *FT* regulation. To validate this hypothesis, two deletion constructs were generated (Figure 5A). The 1.0- and 1.8-kb *FT* promoters were independently fused to GUS to generate various *pFT:GUS* constructs, and they were stably transformed into Col-0 using *Agrobacterium*. GUS-staining assays in *Arabidopsis* showed that the strong GUS signals were induced by both 1.0- and 1.8-kb sequences upstream of cotton *FT*. Furthermore, the activity level of the GUS reporter induced by the 1.0-kb transgenic plants was stronger than in the 1.8-kb transgenic plants; however, there was no difference in the GUS signal induced by the A and D subgenomes’ 1.0-kb *FT* promoter fragments (Figure 5B). A gene transcription analysis using quantitative real-time PCR (qRT-PCR) showed that the expression levels of *GUS* induced by both the 1.0- and 1.8-kb *FT* promoters were consistent with the observed GUS signaling levels (Figure 5C).

### 2.6. Functional Analysis of the 1.8-kb Sequence Upstream of the FT Promoter in Cotton

**Figure 6:**
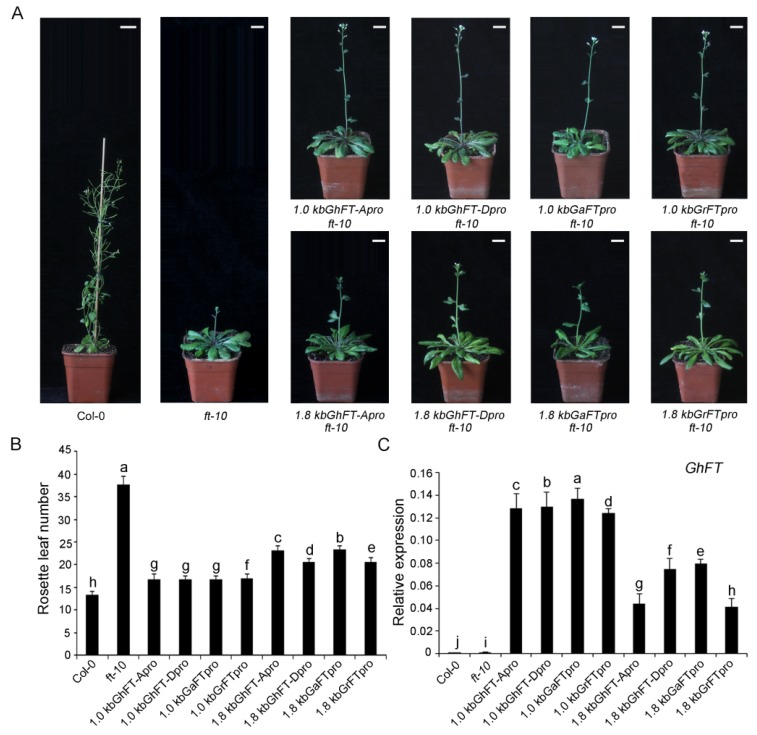
Functional analysis of the 1.8-kb sequence upstream of the *FT* promoter of *G. hirsutum*, *G. arboretum*, and *G. raimondii* using *Arabidopsis* as a model plant. (**A**) Appearance of 30 d Col-0, *ft-10*, *FTpros:GhFT* transgenic *Arabidopsis* lines grown under LD conditions. Scale bar, 2 cm. (**B**) Flowering time was measured as the rosette number of leaves per plant. (**C**) The expression level of *GhFT* was determined by qRT-PCR. Data represent the mean value obtained from three biological replicates. Statistically significant differences are indicated by different lower-case letters (*p* < 0.05, Duncan’s multiple range tests).

To further determine whether regulatory elements exist in the 1.8-kb sequence upstream of the cotton *FT* translation start site, we next generated transgenic *ft-10* mutant *Arabidopsis* harboring 1.0- or 1.8-kb promoter fragments fused to the cDNA of *FT*. Consistent with the reporter gene assays, complementation analyses revealed that these constructs could partially rescue the late-flowering phenotype of *ft-10* plants grown under LD conditions, and the 1.0-kb *FT* promoter fragments were more efficient in rescuing the late-flowering phenotype than the 1.8-kb promoter fragments (Figure 6A,B) in transgenic *Arabidopsis*. In addition, gene transcription analyses in *Arabidopsis* using qRT-PCR showed that the *GhFT* gene was overexpressed in all the transgenic *ft-10* plants under LD conditions (Figure 6C). The data demonstrated that the 1.0-kb *FT* promoter fragment might play an important role in the activity levels of *FT* promoters in cotton, but the possibility of other regions having specific transcriptional regulatory functions was not excluded.

## 3. Discussion

Cotton is an important economic crop, and upland cotton is the most widely planted species in the world [35]. A whole genome-wide analysis showed that there was only one homoeologous *FT* gene pair in the cotton genome, and the *FT*-homologue from *G. hirsutum* promotes early flowering in *A. thaliana* and *Nicotiana tabacum*, providing a good foundation for studying the molecular mechanisms of flowering regulation in cotton [24,25,28,36,37,38]. In this study, we studied the promoters of *GhFT* from the A and D subgenomes of *G. hirsutum*, *GaFT* from the A_2_ genome of *G. arboreum* and *GrFT* from the D_5_ genome of *G. raimondii*.

### 3.1. Cotton FT Promoter Activity Levels Vary with Length and Origin

The promoter functions of the *FT* gene have been studied in several important crops, such as soybean [21], rice [22], and wheat [23], and the results suggested that the promoter activity varies among the different species. In this study, we identified the 1.8-kb promoters of *GhFT-A*, *GhFT-D*, *GaFT*, and *GrFT*, and a phylogenetic analysis classified the four promoters into two types, which corresponded to their genomic origins (Figure 3). A multiple sequence alignment revealed five distinct deletion fragments in the 5.9-kb cotton promoter (Figure 1). A large number of putative plant *cis*-elements were located in the proximal cotton end, such as the TATA-box and CAAT-box, for transcription initiation. In addition, some elements were found to be associated with hormones, light, stress tolerance, tissue expression, and circadian rhythm regulation (Tables S1 and S2).

The binding site CCAAT box of CO and NF-Y transcription factor are located approximately 5.3-kb upstream of *FT* in *Arabidopsis*, whereas the CORE1 and CORE2 sites are located between −220- and −161-bp upstream of the start codon [12]. Functional analyses of the *Arabidopsis FT* promoter indicated that the distal CCAAT element and the proximal CORE sites can regulate *FT* gene expression to control flowering transition. In this study, we analyzed the CORE elements and CCAAT domain of the cotton 1.8-kb *GhFT* promoter and found that they are all located 1.0-kb upstream of the promoter (Figure S1A,B), indicating that the 1.0-kb cotton *FT* promoter may contain the region responsible for activity, but the roles of other regions were not excluded. The CORE elements and CCAAT domain play key roles in the activation of the *FT* promoter in *Arabidopsis*, and the promoter has no activity if any of these motifs are deficient [12,16,18]. In addition, a number of repetitive fragments were found between the 1.1 and 1.4 kb in the *GhFT* promoters (Figure 4), which is an area that contained a large number of *cis*-regulatory elements. We hypothesized that this region may be a binding region of some unknown transcription factors and may also have an effect on the activity of the *FT* promoter. Therefore, we truncated the 1.8-kb *FT* promoter to 1.0 kb (Figure 5A).

GUS histochemical staining assays showed that the GUS activities induced by the 1.0-kb promoters of *GhFT-A*, *GhFT-D*, *GaFT*, and *GrFT* were stronger than their respective 1.8-kb promoters (Figure 5B,C), which indicated that the TGTG(N2-N3)ATG, and CCAAT domains might also determine the activity of the cotton *FT* promoter. Our study also demonstrated that there was no significant difference in promoter activities between the 1.0- or 1.8-kb promoter fragments of the A and D subgenomes using deletion constructs experiments. The promoter activity in the 1.8-kb fragment was weaker than that in the 1.0-kb fragment, implying that some unknown silencers may exist in the proximal cotton *FT* promoter region (−1.0 to −1.8 kb). A large number of repetitive sequences between 1.1- and 1.4-kb upstream of the *FT* translation start site were found (Figure 4), and we speculated that some unknown silencers are probably located in the repetitive enrichment region, which warrants further investigation.

Genetic complement assays showed that the 1.0-kb promoters of *FT* were more efficient in rescuing the late-flowering phenotype of *ft-10* plants than were the 1.8-kb promoters (Figure 6A), with similar rosette leaf numbers being obtained (Figure 6B). Furthermore, the bioinformatics analyses showed that the CORE and CCAAT domains existed in the 1.0-kb promoter of *GhFT*. Based on these experimental results, we speculate that the specific regulation of flowering transition, which involves CO and NF-Y, may exist in this region, but the influences of other regions were not excluded.

### 3.2. Photoperiod Sensitivities of the Promoter Regions from FT Homologues in Arabdiopsis and Cottons Are Different

In *Arabidopsis*, under LD inductive conditions, transcriptional activation of *FT* is directly mediated by CO, which has two responsive elements, CORE1 and CORE2, in the *FT* promoter that share the consensus sequence TGTG(N2-3)ATG [1,9]. The CCAAT-box binding site of CO and NF-Y transcription factors exist approximately 5.3-kb upstream of *FT* in *Arabidopsis*. Rice is a short-day (SD) plant, and the CCAAT box of the potential *cis*-element in the *Hd3a* promoter did not contain any nucleotide changes compared with in *Arabidopsis* [11,22]. The rice *Hd1* gene, which is an orthologue of the *Arabidopsis* CO gene, suppresses the transcriptional activation of *Hd3a* and inhibits flowering under LD conditions. In contrast, *Hd1* gene activates *Hd3a* expression, causing the promotion of flowering under SD conditions [15,39]. Wild cotton is a perennial plant, and most species respond to short day-photoperiods, resulting in a variety of plant types and different flowering time. However, domesticated cotton varieties have undergone extensive artificial selection, resulting in their gradual loss in photoperiodism [27]. Our research revealed that in upland cotton, CORE and CCAAT domains were concentrated in the 1.0-kb region upstream of the translation start site (Figure S1A,B), which was different from in *Arabidopsis* [12]. Compared with the consensus sequence TGTG(N2-3)ATG, the CORE in the cotton *FT* promoter is atypical TGTG(N2-3)ATG. We speculate that CO homolog may bind to the atypical TGTG(N2-3)ATG in upland cotton, but it remains to be confirmed in future. However, whether the FT/CO regulatory model in upland cotton is conserved, as in *Arabidopsis*, remains to be further studied.

## 4. Materials and Methods

### 4.1. Plant Materials and Growth Conditions

*G. hirsutum* cv. Xinluzao 42, *G. raimondii*, and *G. arboreum* were field-grown during the summer of 2016 in Shihezi (Xinjiang, China) under natural conditions. The seeds of the mutant *ft-10* (in the Col-0 background) were ordered from the Arabidopsis Biology Resources Center (ABRC, Columbus, OH, USA). The methods of seed sterilization and cultivation for *ft-10* and *A. thaliana* Col-0, as well as the seedling growth conditions, used in this study were published previously [23,24]. For gene expression analyses, the fresh leaves of Col-0, *ft-10*, and the transgenic lines growing for 20 d were harvested under LD conditions. All the samples were frozen immediately in liquid nitrogen and stored at −80 °C until used.

### 4.2. Phylogenetic Analysis

The genomic sequences of *FTs* from *G. hirsutum*, *G. arboretum*, and *G. raimondii* were assembled using a batch Basic Local Alignment Search Tool (BLAST) search of shotgun sequences obtained from the National Center for Biotechnology Information (http://www.ncbi.nlm.nih.gov). mVISTA was used to perform pairwise alignments (http://genome.lbl.gov/vista) [40,41]. The alignment of the nucleotide sequences and the phylogeny reconstruction analysis of the *FT* promoter in cotton were performed as previously described [42,43,44].

### 4.3. Cloning of the FT Promoter, Vector Construction, and Transformation of Arabidopsis

The cotton *FT* promoter, extending approximately 1.0- and 1.8-kb upstream of the ATG, was cloned by PCR amplification using gene-specific primers designed based on the putative sequences in the *G. hirsutum*, *G. arboreum*, and *G. raimondii* genome database (Table S3) and then ligated to the *pJET 1.2/blunt* vector following the manufacturer’s instructions. The PlantCare database (http://bioinformatics.psb.ugent.be/webtools/plant-care/html/) was used to identify potential *cis*-regulatory elements. To analyze the RSs of the *FT* promoters, we used the Nucleic Acid Dot Plots website (https://en.vectorbuilder.com/tool/nucleic-acid-dot-plot.html) and entered the promoters as required. In the resulting figure, repetitive sequences were represented by point-intensive areas and were calculated according to the values on the coordinate axis. Then, the corresponding *FT* promoters, which were corrected by sequencing, and the *GhFT* gene of *35S:FT* [24] were digested by restriction enzymes to independently replace the *CaMV 35S* promoter and *GUS* gene of pCAMBIA 1301 to generate *pGhFT:FT* constructs. The resulting constructs were transformed into *Agrobacterium tumefaciens* GV3101, which was used to transform Col-0 and *ft-10*, as previously described [45]. Kanamycin-resistant transformants were selected, and homozygotes were replanted and subsequently monitored for flowering using non-transgenic *ft-10* seedlings as controls. Flowering time was assessed using the number of rosette leaves per plant at the time of the first flower bloomed.

To evaluate the activity of the *GhFT* promoter, *pGhFT:GUS* was constructed and transformed into *A. thaliana* (Col-0) to generate independent transgenic lines. The activities of *GhFT* promoters were detected by histochemical GUS staining. Fresh plant tissues were harvested and immersed in GUS staining solution [0.1 M Na_2_HPO_4_·12H_2_O, 0.1 M NaH_2_PO_4_·2H_2_O, pH 7.0, 0.1% Triton X-100, 5 mM K_3_[Fe(CN)_6_], 5 mM K_4_[Fe(CN)_6_]·3H_2_O, 10 mM EDTA-Na_2_, pH 7.0 and 1 mM 5-bromo-4-chloro-3-indolyl-b-D-glucuronic acid]. After incubation at 37 °C overnight, the plant materials were rinsed with 70% ethanol. The resulting stained tissues were observed under a microscope.

### 4.4. Gene Expression Analysis

Total RNA for each sample was isolated using the RNAprep pure Plant Kit (Tiangen, Beijing, China) and treated with RNase-free DNase according to the manufacturer’s protocol. The quality, quantity, and integrity of the total RNA extracted were assessed as previously described [24]. cDNA was synthesized from 200 ng RNA using the Superscript First-Strand Synthesis System (Invitrogen, Carlsbad, CA, USA) according to the manufacturer’s protocol. qRT-PCR was carried out using the SYBR Green Master Mixture (CWBIO, Beijing, China) on an Applied Biosystems 7500 Fast Real-Time PCR System (Life Technologies, Foster City, CA, USA). The PCR amplification system and program were as described previously [14]. Three biological replications were performed with independently isolated RNA in all the qRT-PCR assays. Relative gene expression levels were calculated using the 2^–ΔΔCt^ method [46].

### 4.5. Statistical Analysis

The results were based on three independent experiments with at least three replicates. The SPSS software package (ver.17.0; SPSS Inc., Chicago, IL, USA) was used for the statistical analysis, as previously described [47].

## 5. Conclusions

In this study, we identified a 5.9-kb promoter of *FT* from *G. hirsutum* and revealed that the activity level of the truncated *GhFT* promoter varied in different tissues. Further studies on the 1.0- and 1.8-kb fragments of the *FT* promoters in *G. hirsutum*, *G. arboreum*, and *G. raimondii* showed that the activity of the 1.0-kb *FT* promoter was higher than that of the 1.8-kb promoter. These results illustrate that the proximal *FT* promoter fragment might play an important role affecting the activity levels of *FT* promoters in cotton flowering.

## Supplementary Materials

Supplementary materials can be found at https://www.mdpi.com/1422-0067/20/19/4769/s1.
