# Supplementary material for: Characterization and Activity Analyses of the FLOWERING LOCUS T Promoter in Gossypium Hirsutum
Source: Int J Mol Sci. 2019 Sep 26;20(19):4769. doi: 10.3390/ijms20194769 (PMC6801411; doi:10.3390/ijms20194769)
Supplement: Supplementary file 1 [file ijms-20-04769-s001.pdf]

Table S1. Prediction of *cis*-regulatory elements in the 1.8-kb *GhFT-A* promoter fragment

Site name	Location	Positive or negative sequence	Domain sequence	Function
TATC-box	-1611	-	TATCCCA	*cis*-acting element involved in gibberellin responsiveness
G-box	-1571	-	CACACATGGAA	involved in light responsiveness
Pc-CMA2c	-1566	-	GCCCACACA	part of a light responsive module
Box 1	-1416/-1053/-744	+/+/+	TTTCAAA	involved in light responsiveness
as-2-box	-1330/-1148/-908	+/+/-	GATAATGATG	involved in shoot-specific expression and light responsiveness
Box 4	-1308	+	ATTAAT	part of a light responsive module
ABRE	-1259	-	TACGGTC	*cis*-acting element involved in the abscisic acid (ABA) responsiveness
ACE	-1241	-	AAAACGTTTA	involved in light responsiveness
AT1-motif	-1110	-	ATTAATTTTACA	part of a light responsive module
TCA-element	-935	-	CAGAAAAGGA	*cis*-acting element involved in salicylic acid responsiveness
P-box	-934	+	CCTTTTG	*cis*-acting element involved in gibberellin responsiveness
HSE	-861	+	AAAAAATTTC	*cis*-acting element involved in heat stress responsiveness
CAT-box	-780	-	GCCACT	*cis*-acting regulatory element related to meristem expression
MBS	-742/-417	+/-	CAACTG	MYB Binding Site
5'UTR Py-rich stretch	-620	-	TTTCTTCTCT	*cis*-acting element conferring high transcription levels
TC-rich repeats	-618	-	ATTTTCTTCA	*cis*-acting element involved in

				defense and stress responsiveness
GT1-motif	-608/-606	+/-	GGTTAA	involved in light responsiveness
Sp1	-410/-277	-/+	CC(G/A)CCC	involved in light responsiveness
circadian	-153	-	CAANNNNATC	*cis*-acting regulatory element involved in circadian control
CAAT-box	-147/-72…	-/-	CAAT	common *cis*-acting element in promoter and enhancer regions
TATA-box	-100	+	TATA	core promoter element around -30 of transcription start
ARE	-57/-49	-/-	TGGTTT	*cis*-acting regulatory element essential for anaerobic induction
TCT-motif	-21	-	TCTTAC	part of a light responsive module

Table S2. Prediction of *cis*-regulatory elements in the 1.8-kb *GhFT-D* promoter fragment

Site name	Location	Positive or negative sequence	Domain sequence	Function
ATCT-motif	-1722	+	AATCTAATCT	part of a light responsive module
Box 4	-1711	+	ATTAAT	part of a light responsive module
TATC-box	-1672	-	TATCCCA	*cis*-acting element involved in gibberellin responsiveness
GC-motif	-1622	+	CCCCCG	Hypoxia induced response element
Sp1	-1619/-410 /-277	-/-/+	GGGCGG/CC(G/A) CCC	involved in light responsiveness
as-2-box	-1575/-138 8/-1208	+/+/+	GATAatGATG	involved in shoot-specific expression and light responsiveness
ACE	-1482/-130 0/-1120	-/-/-	AAAACGTTTA	involved in light responsiveness
AuxRR-CORE	-1381	-	GGTCCAT	involved in auxin response due to

				stress
ABRE	-1249/-1171/-1067	-/-/-	TACGGTC	*cis*-acting element involved in the abscisic acid (ABA) responsiveness
G-Box	-1249/-1067	+/+	CACACATGGAA	involved in light responsiveness
MBS	-1174	-	CAACTG	MYB Binding Site
Box 1	-1113/-775	+/+	TTTCAAA	involved in light responsiveness
P-box	-949	+	CCTTTTG	*cis*-acting element involved in gibberellin responsiveness
TCA-element	-950	-	CAGAAAAGGA	*cis*-acting element involved in salicylic acid responsiveness
HSE	-862	+	AAAAAATTTC	*cis*-acting element involved in heat stress responsiveness
CAT-box	-787	-	GCCACT	*cis*-acting regulatory element related to meristem expression
5'UTR Py-rich stretch	-622	-	TTTCTTCTCT	*cis*-acting element conferring high transcription levels
TC-rich repeats	-620	-	ATTTTCTTCA	*cis*-acting element involved in defense and stress responsiveness
GT1-motif	-610/-608	+/-	GGTTAA	involved in light responsiveness
circadian	-153	-	CAANNNNATC	*cis*-acting regulatory element involved in circadian control
CAAT-box	-147/-72	-/-	CAAT	common *cis*-acting element in promoter and enhancer regions
TATA-box	-100	+	TATA	core promoter element around -30 of transcription start
ARE	-57/-49	-/-	TGGTTT	*cis*-acting regulatory element essential for anaerobic induction
TCT-motif	-21	-	TCTTAC	part of a light responsive module

Table S3. PCR primers used in this study

Primer	Primer Sequence (5'-3')	Function
Pst I-1.0k GhFT*p*-F	CGCTGCAGCATCCTAATCCCTTTCCC	clone of the 1.0-kb promoter in upland cotton
Pst I-*1.5k GhFTp-F*	CGCTGCAGTTACACGAAATATCAT	clone of the 1.5-kb promoter in upland cotton
Pst I-1.8k GhFT*p*-F	GGCTGCAGTGAATGACTCCTCCTCAG	clone of the 1.8-kb promoter in upland cotton
Pst I-4.2k GhFT*p*-F	CGCTGCAGGCGATACCAAAGGCAGTC	clone of the 4.2-kb promoter in upland cotton
Pst I-*4.8k GhFTp*-F	CGCTGCAGCCTCCACTCACTAATCCC	clone of the 4.8-kb promoter in upland cotton
Pst I-5.9k GhFT*p*-F	CGCTGCAGGAAATCAACTTCCCCACTT	clone of the 5.9-kb promoter in upland cotton
Nco I-*GhFTp*-R	CGCCATGGTAACCCTACCAACAACCAA	clone of the promoter in upland cotton
Pst I-*1.8k FT-Dp*-F	GGCTGCAGTGAATGACTCCTCCTCAG	clone of the 1.8-kb promoter of D subgenome in cotton
Pst I-*1.8k FT-Ap*-F	GGCTGCAGTGAATTACTCCTCCTCAT	clone of the 1.8-kb promoter of A subgenome in cotton
Nco I-*FTp*-R	CGCCATGGGATATCGCTATTTGGTCTTAC	clone of the promoter in cotton
Pst I-*1.0k FT-Dp*-F	CGCTGCAGATCATAATCCCTTTCCCCTT	clone of the 1.0-kb promoter of D subgenome in cotton
Pst I-*1.0k FT-Ap*-F	CGCTGCAGATCCTAATCCCTTTCCCCTC	clone of the 1.0-kb promoter of A subgenome in cotton
Nco I-*FTp*-R	CGCCATGGGATATCGCTATTTGGTCTTAC	clone of the promoter in cotton
qGUS-F	TTCGATGCGGTCACTCATTA	qRT-PCR
qGUS-R	TAGAGCATTACGCTGCGATG	
qGhFT-F	TCTGCTATGAGAGCCCACGA	qRT-PCR
qGhFT-R	TCATGTCCTACGGCCACCGGATCCACT	
actin2-F	ATACGTGCAACAAACCC	qRT-PCR
actin2-R	CTACCTCCCCGTGTCA	

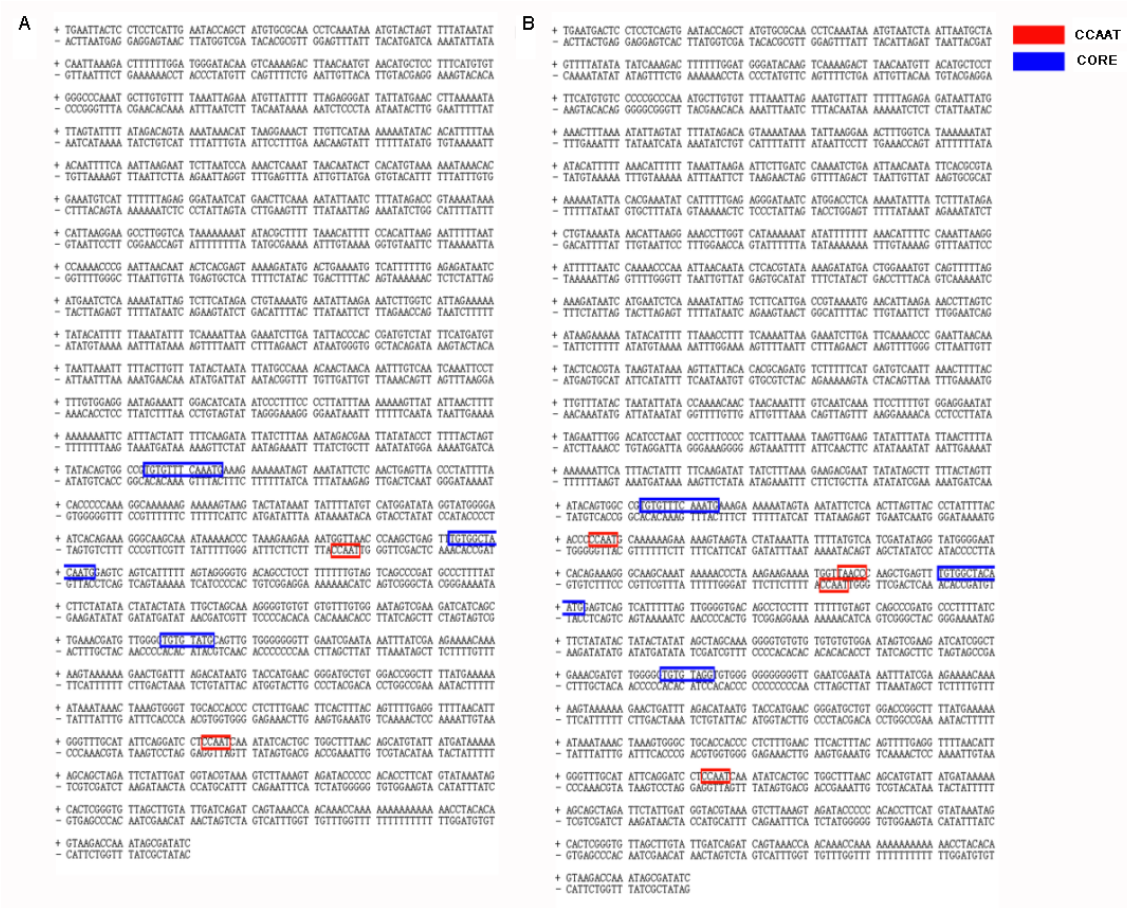

Figure S1. CORE and CCAAT domains in the 1.8-kb cotton *FT* promoters. (A) *GhFT-A* promoter. (B) *GhFT-D* promoter. The red box represents the CCAAT domain. The blue box represents the CORE domain.
